# Pilon fracture with central articular surface collapse treated by posterior tibial fenestration indirect reduction and compression technique: Two cases report

**DOI:** 10.1097/MD.0000000000039151

**Published:** 2024-08-16

**Authors:** Chaohui Sang, Langqing Zeng, Huafeng Zhong, Qiang Wang, Lulu Zeng

**Affiliations:** a Yixing People’s Hospital and Department of Orthopaedic Center, Wuxi, Jiangsu, China; b Zhuhai People’s Hospital (Zhuhai Clinical Medical College of Jinan University) and Department of Orthopaedics, Zhuhai, Guangdong, China; c Zhuhai People’s Hospital (Zhuhai Clinical Medical College of Jinan University) and Department of Anesthesiology, Zhuhai, Guangdong, China; d Zhuhai People’s Hospital (Zhuhai Clinical Medical College of Jinan University) and Department of Nursing, Zhuhai, Guangdong, China.

**Keywords:** central articular surface reduction, intact anterolateral cortex, pilon fracture, posterior tibial fenestration indirect reduction and compression

## Abstract

**Rationale:**

Central collapsed fracture blocks traditionally require either an anteromedial or anterolateral approach for reduction. However, existing techniques face challenges such as soft tissue damage and compromised tibial strength, especially in pilon fractures with central articular surface collapse and an intact anterior cortex, as classified under 43B2.3 in the 2018 Orthopaedic Trauma Association/Association for the Study of Internal Fixation Fracture and Dislocation Classification Compendium.

**Patient concerns:**

We address the management of pilon fractures with central articular surface collapse, focusing on 2 cases where conventional reduction techniques posed a risk to soft tissues and tibial integrity.

**Diagnoses:**

The patients presented with pilon fractures characterized by a central articular surface collapse and an intact anterior cortex, aligning with the 43B2.3 classification.

**Interventions:**

A novel approach was employed, utilizing posterior tibial fenestration and indirect reduction with compression techniques. This method leveraged the talus as a template for precise articular surface realignment.

**Outcomes:**

Both cases demonstrated excellent reduction of the distal tibial articular surface and achieved favorable functional recovery of the ankle, evidenced by high American Orthopedic Foot and Ankle Society Ankle Hindfoot Scale scores during the 3-year follow-up.

**Lessons:**

The posterior tibial fenestration technique offers significant advantages for distal tibial pilon fracture reduction. It allows for precise articular realignment, facilitates bone grafting, and minimizes soft tissue and cortical bone disruption. This method is particularly effective for pilon fractures with an intact anterolateral cortex and central articular collapse, providing a valuable surgical alternative.

## 1. Introduction

Pilon fracture refers to a distal tibial fracture involving the tibial articular surface due to axial violence, accounting for only 5% to 7% of all tibial fractures.^[1,2]^ In the 2018 Orthopaedic Trauma Association/Association for the Study of Internal Fixation (AO) Fracture and Dislocation Classification Compendium, a new classification, 43B2.3, was introduced to encompass pilon fractures with central articular surface collapse.^[3]^

Reduction of the central collapsed fracture block is conventionally achieved through either an anteromedial or anterolateral approach, wherein the anterior bone block is elevated to directly visualize and reinstate the central articular surface.^[4]^ However, the current method may prove insufficient in addressing pilon fractures characterized by central collapse of the articular surface and an intact anterior cortex (43B2.3).

We found that for this type of pilon fracture, posterior tibial fenestration, indirect reduction, and compression techniques could be used to better reset the distal tibial articular surface without expanding the incision. This paper summarizes 2 cases of patients who used this method to reset this type of pilon fracture with satisfactory clinical follow-up. The detailed surgical approach and clinical significance are described here.

## 2. Case report

### 2.1. Case 1

The patient was a 48-year-old male who fell from a height of 3 m and complained of left ankle pain. Physical examination revealed swelling, deformity, tenderness, and bone crepitus in the left ankle with impaired mobility. Vascular perfusion, sensory perception, and distal toe mobility were normal.

**Figure 1.**
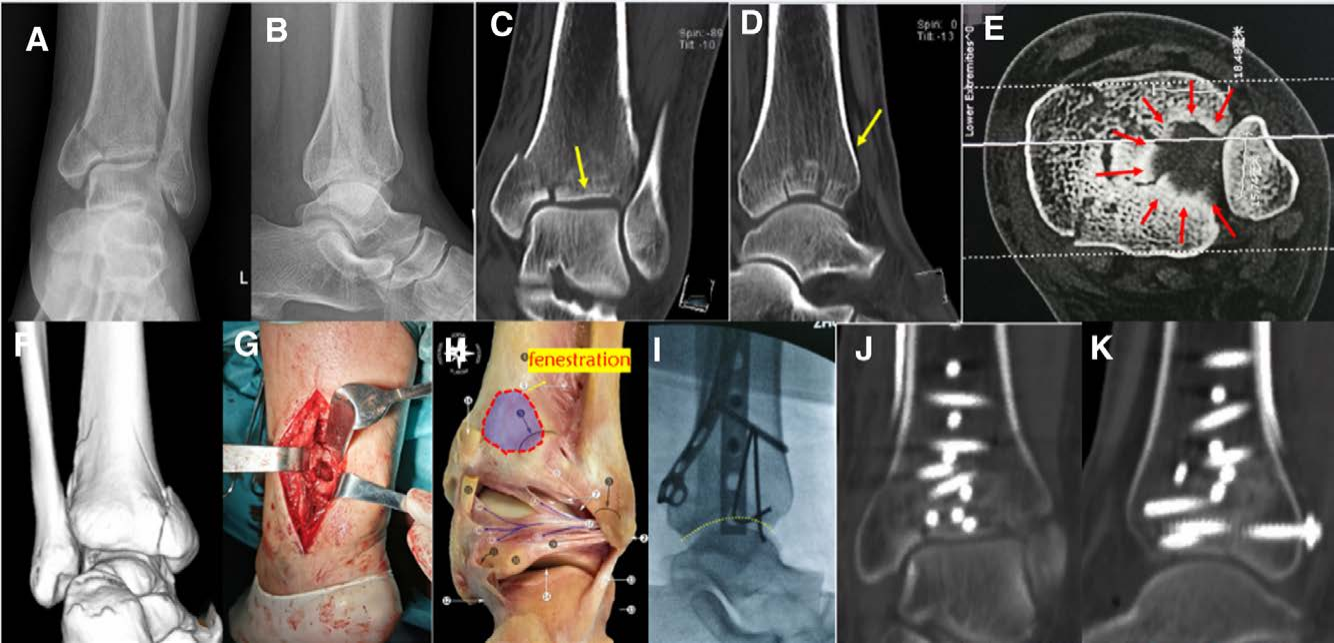
Frontal and lateral X-rays of the left ankle upon admission to the hospital (A and B). CT scan revealed a comminuted fracture involving the inner ankle, central region, and posterior lateral aspect of the distal tibia, with intact anterolateral bone cortex (C–F). Intraoperative windowing beyond the posterior articular surface of the distal tibia (G and H). The distal articular surface of the tibia was well reduced after posterior tibial fenestration indirect reduction and compression (I). The postoperative CT scan revealed satisfactory reduction of the articular surface, with central collapse (J and K). CT = computed tomography.

X-ray and computed tomography (CT) scans confirmed a comminuted fracture of the distal left tibia, primarily in the inner ankle, central, and posterior lateral regions, with an intact anterolateral bone cortex (Fig. 1A–F). The admission diagnosis was left distal tibial fracture (AO 43B2.3). The patient’s ankle was initially immobilized in a plaster cast and elevated for 1 week to reduce swelling. The surgical incision strategy involved a combination of the posterior lateral incision and medial approach with minimally invasive plate osteosynthesis, as the patient’s fracture mass was primarily located in the medial ankle, central, and posterior lateral regions, while the anterolateral cortex remained intact. The surgical procedure was as follows.

A medial minimally invasive plate osteosynthesis incision was implemented, accompanied by the utilization of a 1/3 tubular plate to immobilize the medial block.

The posterolateral approach was used to expose the posterior tibia. A meticulous window, approximately 2 to 3 cm proximal to the articular surface was created. The talar surface served as the template. Indirect compression using a periosteal stripper and subsequent resetting of the collapsed articular surface bone block were performed (Fig. 1G and H).

The resulting bone defect caused by the reset of the proximal tibia was addressed by introducing allograft bone.

The fracture block was effectively stabilized using a posterior lateral plate, ensuring precise realignment of the distal tibial articular surface with the aid of C-arm fluoroscopy during the surgery (Fig. 1I).

Postoperative assessment confirmed satisfactory alignment (Fig. 1J and K) and fracture consolidation occurred within 4 months. Over a 3-year follow-up period, the patient exhibited significant recovery, as evidenced by a favorable American Orthopedic Foot and Ankle Society Ankle-Hindfoot Scale score of 92.

### 2.2. Case 2

**Figure 2.**
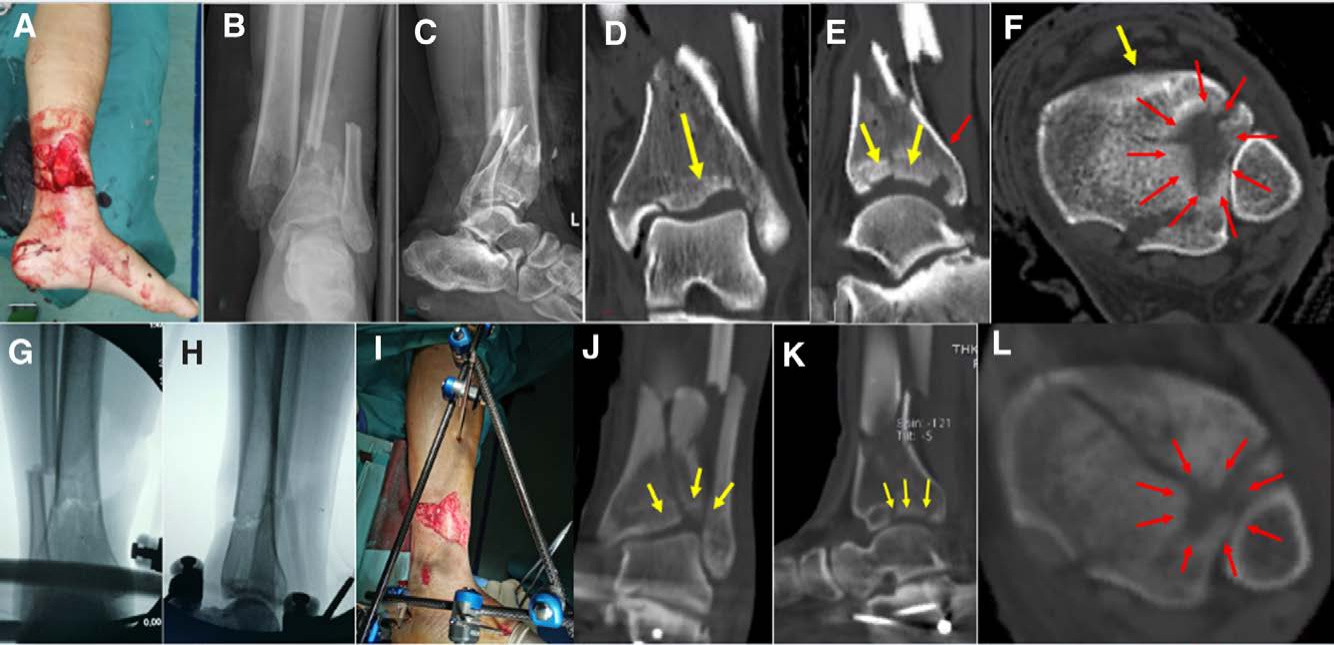
The left ankle in case 2 had an open fracture with the broken end exposed (A). The X-ray showed a comminuted fracture of the left distal tibiofibular (B and C). CT scan showed a comminuted fracture of the left distal tibia and fibula with collapse of the central articular surface of the distal tibia and an intact anterior cortex (D–F). The broken end of the fracture was fixed with an external fixator during surgery, and fluoroscopy confirmed good recovery of the force line (G and H). Postoperative CT showed that the articular surface was partially repositioned, but the central region was still partially collapsed (J–L). CT = computed tomography.

Another 48-year-old male patient was admitted to the hospital with left ankle pain and limited mobility for 2 hours due to a fall from a high place. Examination revealed an 8 * 6 cm wound along the anteromedial edge of the lower left calf, exposed bone, deformity, and oozing blood (Fig. 2A). The left dorsalis pedis artery showed diminished pulsation and the heel region displayed swelling, tenderness, and weaker sensory perception.

Radiography and CT confirmed a comminuted distal tibiofibular and calcaneal fracture on the left side (Fig. 2B–F). Admission diagnosis: 1. left distal tibial fracture (AO typing 43B2.3), Gustilo 3A open fracture; 2. left distal fibular fracture, 3. left calcaneal fractures. A staged surgical approach was adopted because of the open fracture, involving trauma debridement, temporary fixation with external fixation, and proper alignment through intraoperative C-arm fluoroscopy (Fig. 2G–I). Percutaneous Kirschner wires addressed calcaneus fixation, and vacuum-assisted closure managed the open wound. Postoperative CT showed that the articular surface was partially repositioned, but the central region was still partially collapsed (Fig. 2J–L).

**Figure 3.**
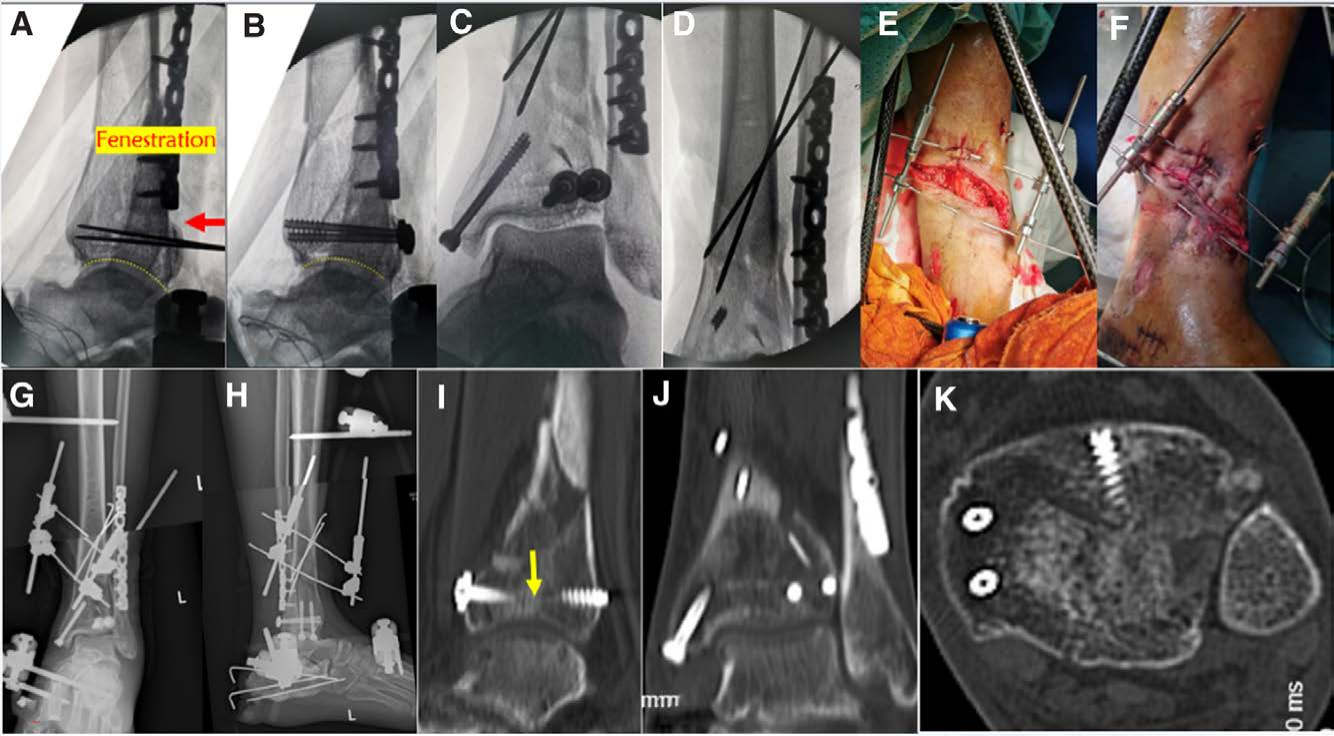
The distal articular surface of the tibia was well reduced after posterior tibial fenestration indirect reduction and compression (A and B). External fixation combined with Kirschner pin for fixation of distal tibia fracture (C and D). Closure of medial tibial wounds using skin retractors (E and F). The postoperative X-ray revealed a well-aligned fracture end force line and reduced articular surface (G and H). CT scan at 6 weeks postoperative showed that the articular surfaces were well repositioned and aligned (I–K). CT = computed tomography.

The second stage of surgery was performed 1 week later. The posterolateral approach was used. First, internal fixation with plate screws was performed after reduction of the fractured fibula end. Subsequently, the posterior cortex of the tibia was exposed to indirectly reset the articular surface fracture block by creating a window in the posterior cortex of the distal tibia using a periosteal peeler (Fig. 3A). Reconstruction of the fracture site by filling the bone defect with the graft material. Finally, cannulated lag screws were used to fix the posterior block (Fig. 3B). Medial ankle fractures were addressed using percutaneous cannulated lag screws (Fig 3C and D). An anteromedial wound retractor was gradually employed to close the traumatic site while maintaining external fixation (Fig 3E and F). Postoperative radiography of the left ankle demonstrated satisfactory alignment of the fracture fragments (Fig. 3G and H). A CT scan at 6 weeks postoperatively showed that the articular surfaces were well repositioned and aligned (Fig. 3I–K).

The 3-year follow-up demonstrated successful fracture union and improved ankle range of motion and ambulatory function, with an American Orthopedic Foot and Ankle Society Ankle-Hindfoot Scale score of 86.

## 3. Discussion

Pilon fractures are often caused by high-energy injuries and are characterized by compression, comminution, a high degree of instability of the tibial metaphysis, uneven articular surfaces, and damage to the articular cartilage. They are often associated with concomitant polytrauma with the presence of open wounds, degloving injuries, and severe soft tissue trauma.^[5,6]^

The 2018 revision of the AO fracture classification introduced a new subtype, 43B2.3, which is referred to as the central-type articular surface collapse in pilon fractures. This addition complements the existing classifications of distal tibial fractures, specifically 43B2.1 and 43B2.2. Unlike previous fracture types that only partially impacted the central articular surface, this subtype is distinguished by experiencing a direct force leading to articular surface collapse and compression. Additionally, it is accompanied by fractures of the cortical bone, distant from the site of articular surface collapse.^[3]^

In 1979, Rüedi and Allgöwer proposed that one of the principles of pilon fracture treatment is anatomic reduction of the articular surface.^[7]^ The following methods are commonly used to reset articular surfaces:

Method 1 employs external fixation for patients with severe soft tissue injuries around the ankle who are unable to undergo an incision-based articular surface bone block reset.^[8]^ By distracting the ankle space, this method indirectly resets the articular surface bone block through ligament and joint capsule tension, minimizing damage to the surrounding tissues. However, a significant limitation is the inability to achieve a direct anatomical reduction of the articular surface.^[9]^ Furthermore, central fracture blocks lacking ligament and joint capsule attachments may pose challenges for effective reset using this technique.

Method 2 focused on the comminuted distal tibia in pilon fractures, prioritizing reduction of the rarely comminuted fibula. Restoring the fibular length indirectly reduces the Volkmann and Chaput blocks of the distal tibia through tension in the anterior and posterior inferior tibiofibular ligaments.^[2]^ The posterolateral Volkmann fracture block serves as a constant bone block and a reference point for reconstructing and resetting the distal tibial articular surface.^[4,9]^

Method 3 involves the anterolateral approach and anterior lifting of the fracture block for direct visualization of the central articular surfaces.^[4]^ Articular surfaces were realigned using a talus template. Disadvantages include increased difficulty in lifting the anterior bone block in patients with a large anterior block or no fracture of the anterior bone cortex, potentially increasing soft tissue damage, and increasing the risk of necrosis and infection.

Method 4 employed an implant rod for superior compression to realign the tibial articular surface. A posterior proximal tibial tuberosity site window was created using the superior talar margin as a template for realignment and filling of the bone defect with grafts. This approach avoids anterior surgery-related cutaneous complications but has limitations, including restricted space for the implant rod, hindered visualization of the articular surface, and the need for an additional surgical incision above the fracture line. This supplementary procedure compromises the bone integrity and increases the risk of surgical trauma. The limited space allows only smaller bone particles, which negatively affects the effectiveness of the bone-grafting procedure.

The anterior approach is suboptimal for visualizing and reducing the articular surface in pilon fractures with central collapse and intact anterior cortex. In this type of fracture, direct visualization of the articular surface through an anterior incision is not possible because of the intact anterior cortex. It is necessary to reset the centrally collapsed fracture block after lifting the bone anterior to the tibia, which not only increases the trauma of the operation but also decreases the strength of the tibia. Moreover, the anterior aspect of the distal tibia is a weak area of soft tissue with poor blood flow and the addition of an anterior incision may increase the risk of skin necrosis and infection.

**Figure 4.**
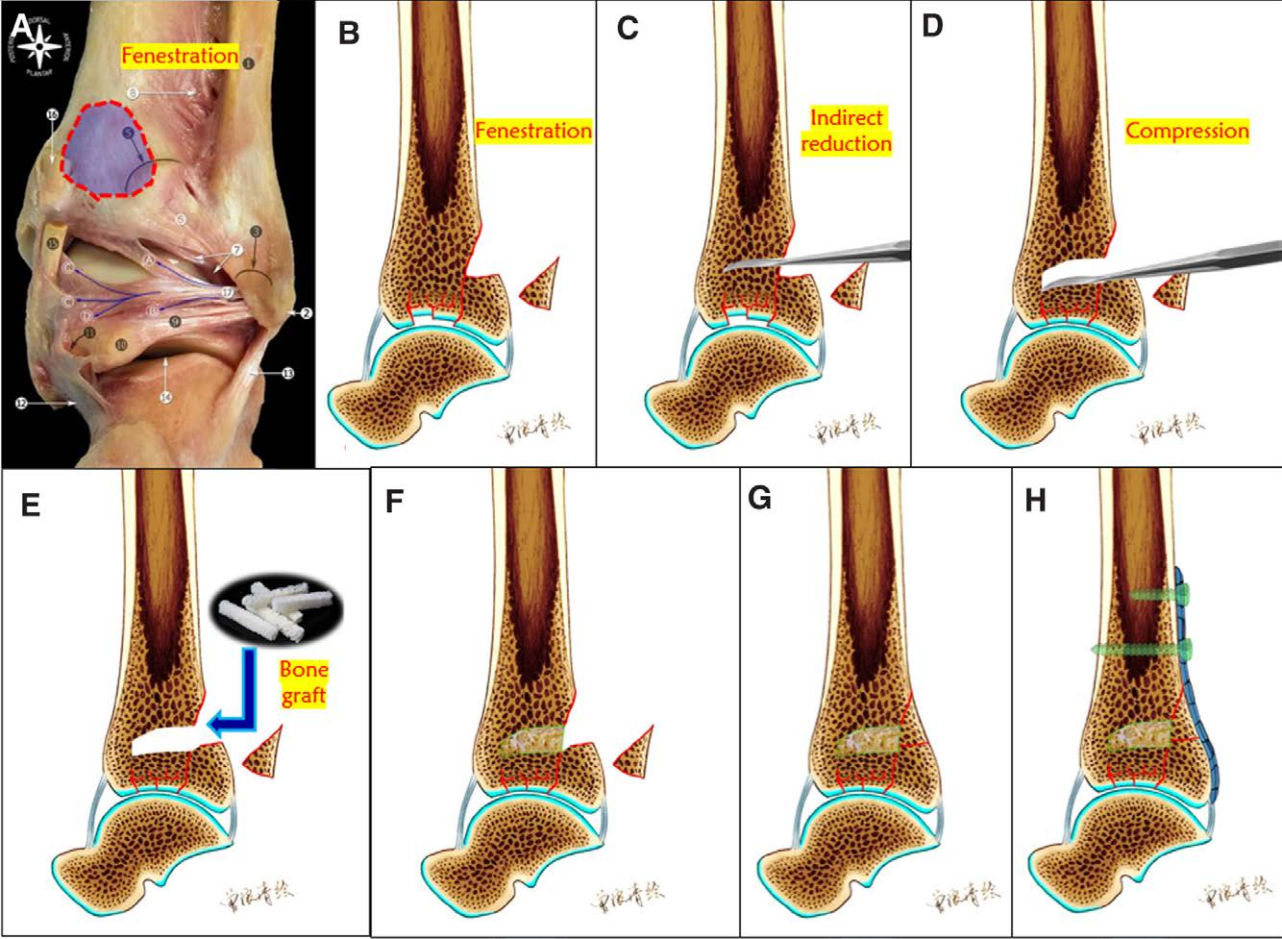
A window is created in the posterior tibia (A and B). Using the superior articular surface of the talus as a template to reset a collapsed central articular surface fracture (C and D). Bone grafting in bone defects (E and F). Using a 1/3 tubular steel plate for buttress support and fixation of both the articular surface and posterior bone block (G and H).

To address these challenges, we made pertinent improvements and developed an indirect reduction and compression technique for posterior fenestration (Fig. 4). This technique is primarily applicable to pilon fractures characterized by a relatively intact anterior cortex and central articular surface collapse.

This technique involves utilizing the posterior lateral approach to expose the posterior tibial cortex. Subsequently, a window was created in the posterior tibia, approximately 2 to 3 cm proximal to the articular surface (Fig. 4A and B). The articular surface above the talus served as a template, and the central collapsed bone was meticulously reset using a periosteal peeler (Fig. 4C and D). Following this step, bone grafting was performed to address any defects (Fig. 4E and F). During surgery, fluoroscopy was employed to realign and reset the articular surface before using a 1/3 tubular steel plate for buttress support and fixation of both the articular surface and posterior bone block (Fig. 4G and H). The proposed method has several advantages.

1. *Improved soft tissue considerations*: The posterior soft tissues demonstrate greater thickness and superior vascularity than those in the anterolateral approach. Resetting the centrally collapsed articular surface using a posterolateral approach mitigates the risk of soft-tissue complications.
2. *Combined fracture management*: The same incision can be used for the reduction and fixation of both fibula fractures and the central articular surface of the tibia, resulting in fewer incisions and reduced soft tissue damage.
3. *Enhanced procedural efficiency*: The key distinction between traditional implant rods and the new design is window positioning. In this new design, the window was located approximately 2 to 3 cm proximal to the distal tibial articular surface and remained within the surgical field of view. This strategic placement facilitates easier access for creating a window, repositioning fractures, and implantation.

The effectiveness of our approach in addressing central articular surface collapse of the distal tibia was validated through postoperative CT evaluations in our 2 patients. In the context of distal tibial fractures, the literature reports a significantly higher incidence of nonunion compared to other long bones.^[10]^ Localized bone loss following distal tibial reduction is a contributing factor, and effective bone grafting can mitigate nonunion rates and prevent reduction loss by providing support to the articular surface.^[2,11]^ Our approach additionally facilitates adequate and effective bone grafting through the surgical window after resetting the articular surface of the distal tibia, thus reducing the risk of postoperative fracture nonunion.

However, a shared limitation of posterior approaches for realizing pilon fractures is the inability to directly visualize the distal tibial articular surface during the realignment process. Relying solely on fluoroscopy to assess reset outcomes during surgery may result in an insufficient realignment.

## 4. Conclusion

In this paper, we report 2 cases of pilon fractures treated with indirect reduction and compression techniques of posterior fenestration. This technique can effectively reset the centrally collapsed bone of a pilon fracture, and the patients’ ankle function recovered well after the operation at the 3-year follow-up. Therefore, we believe that this is an effective technique to reset the centrally collapsed bone of a pilon fracture, and it is even more advantageous for intact anterior cortical pilon fractures with a centrally collapsed articular surface.

## Author contributions

**Conceptualization:** Chaohui Sang, Langqing Zeng.

**Methodology:** Langqing Zeng.

**Project administration:** Chaohui Sang, Langqing Zeng.

**Resources:** Huafeng Zhong.

**Visualization:** Huafeng Zhong.

**Writing – original draft:** Chaohui Sang.

**Writing – review & editing:** Langqing Zeng, Qiang Wang, Lulu Zeng.
